# Risk Factors and Clinical Outcomes of Perioperative Hypotension in the Neck of Femur Fracture Surgery: A Case-Control and Cohort Analysis

**DOI:** 10.7759/cureus.73788

**Published:** 2024-11-15

**Authors:** Kavitha Paul, Ameya Elizabeth Benedict, Sweta Sarkar, Robin R Mathews, Ashwin Unnithan

**Affiliations:** 1 Acute Medicine, Ashford and St Peter's Hospitals NHS Foundation Trust, Chertsey, GBR; 2 Intensive Care Unit, Ashford and St Peter's Hospitals NHS Foundation Trust, Chertsey, GBR; 3 Trauma and Orthopaedics, Ashford and St Peter's Hospitals NHS Foundation Trust, Chertsey, GBR

**Keywords:** geriatric hip fracture, geriatric injuries, intraoperative hypotension, neck of femur fractures, perioperative hypotension, perioperative morbidity, perioperative mortality, perioperative outcomes, perioperative risk factors, postoperative hypotension

## Abstract

Introduction

Neck-of-femur (NOF) fractures have high prevalence rates and require prompt surgical intervention for better outcomes. Perioperative hypotension (POH) in the geriatric population has poor outcomes with several contributing factors. The study intends to explore these risk factors and their correlation with patient outcomes.

Methodology

We studied a total of 276 patients who underwent surgical fixation of the NOF fracture at St Peter’s Hospital, Surrey, from June 1, 2022, to June 1, 2023. Patients with POH were identified; the risk factors and one-year outcome were studied to obtain the results. We used odds ratio (OR), relative risk (RR), and multivariate regression to analyse the statistical association within the data.

Results

The incidence of POH was 68% (188/276) which included preoperative hypotension (9.78%), intraoperative hypotension (48.55%), and postoperative hypotension with a mean arterial pressure (MAP) of <65 mmHg (24.63%) and fall of systolic blood pressure to less than 80% (34.42%). Statistically significant risk factors were hypertension (OR: 1.330), heart disease (OR: 2.768), and hemoglobin (Hg) drop (OR: 1.42). The outcomes we studied were all statistically significant, with an RR of more than one. It includes postoperative delirium (RR: 2.037), postoperative 30-day morbidity (RR: 4.008), postoperative 30-day mortality (RR: 6.12), 365-day mortality (RR: 2.224), postoperative delay in mobilisation (RR: 1.329), and prolonged length of stay (RR: 1.273).

Conclusion

The study shows a clear association between POH and increased postoperative complications, highlighting the need for prompt intervention. This case-control study identified hypertension, history of heart disease, and perioperative blood loss as significant risk factors for developing POH. Also, this study demonstrates that POH is significantly associated with adverse outcomes, including the increased risk of delirium, prolonged hospital stays, and elevated 30-day morbidity in elderly patients undergoing hip fracture surgery. The findings also indicated that the duration of hypotension did not directly influence the outcomes; its occurrence alone is a significant factor in developing these complications.

## Introduction

Hip fractures are complicated injuries that usually affect the elderly population, with profound implications for personal outcomes and rising demands on the healthcare system. According to the National Hip Fracture Database (NHFD), around 76,000 hip fractures occur each year in the UK. With age- and sex-specific incidence rates presumed stable, the global occurrence of hip fractures is projected to surge, potentially reaching 2.6 million cases by 2025 and escalating to 4.5 million by 2050 [1]. The management of hip fractures is complex due to the underlying comorbidities in geriatric population, compounded by potential postoperative complications.

Blood pressure is a complex parameter due to its multifactorial physiology and broad implications on the human body. It is measured precisely during the perioperative period, and any fluctuations are often managed without delay. In the older population, preexisting comorbidities and stress from surgery and anesthesia can cause considerable variations in the perioperative blood pressure, making the management challenging. The reduced physiological reserve in elderly, especially under surgical stress, limits their ability to adapt to the blood pressure fluctuations, further complicating the management [2]. Packiasabapathy et al. illustrates this with an analogy. Similar to how an athletes’ cardiopulmonary reserve allows them to perform better under physical strain, the intricacies during surgery mirror a patient’s physiological reserve and capacity to adjust to stress. This declines with age and makes blood pressure control during the surgery more critical as well [2].

Standardised blood pressure targets during perioperative management are poorly defined, which can be attributed to the multifactorial physiology. The body employs various mechanisms for short-term and long-term blood pressure control, and each organ maintains its own blood flow by autoregulatory mechanism [3]. Individual differences in long-term health conditions and physiology further impact these regulatory mechanisms, highlighting the challenges in defining blood pressure targets [4]. The fundamental goal of perioperative blood pressure management is to ensure adequate organ perfusion and to minimise the risk of organ damage. Uncontrolled blood pressure variations can cause ischemia and other adverse events after the surgery [4].

Previous literature has shown a positive correlation between perioperative hypotension (POH) in hip fracture surgeries and an increased risk of postoperative complications [5-7]. Major adverse events during surgery are a significant concern in these patients, as they contribute to postoperative morbidity, mortality, extended hospital stays, and delayed rehabilitation, all of which impact the quality of life and may pose life-threatening risks.

Moreover, POH following hip fracture surgery poses a significant financial burden on healthcare systems, including the National Health Service (NHS) in the UK. Stapelfeldt et al. showed that patients with POH have higher rates of 30-day readmissions and prolonged postoperative hospital stay [8].

In this study, we examined the potential risk factors and expected outcomes related to POH, with the aim of identifying high-risk patients and providing them with tailored management strategies to enhance immediate and long-term outcomes.

## Materials and methods

Study design

This study was designed as a combined case-control and cohort study to analyse risk factors and outcomes of perioperative hypotension in the neck-of-femur fracture patients undergoing surgery. The study took place at Ashford and St Peter’s Hospital NHS Trust, Surrey, from June 2024 to October 2024. The data were analysed using MS Excel (Microsoft Corporation, Redmond, Washington, United States). This study meets all the requirements outlined in the Strengthening the Reporting of Observational Studies in Epidemiology (STROBE) checklist.

Case-Control Study

The case-control component studied the risk factors for POH in patients undergoing surgery for the neck-of-femur fracture, who were scheduled between June 1, 2022, and June 1, 2023.

Cases: Patients admitted to St Peter’s Hospital within the study period who were diagnosed with a NOF fracture and had experienced perioperative hypotension, defined as either a mean arterial pressure (MAP) of less than 65 mmHg in the immediate preoperative, intraoperative, or postoperative period, or a systolic blood pressure decrease of more than 20% from baseline during surgery for the NOF fracture.

Controls: Patients admitted to St Peter’s Hospital within the study period who were diagnosed with an NOF fracture and did not experience POH, defined as either a MAP of less than 65 mmHg in the immediate preoperative, intraoperative, or postoperative period, or a systolic blood pressure decrease of more than 20% from baseline during surgery for the NOF fracture.

The inclusion criteria for case-control study included the following: patients of any age diagnosed with an NOF fracture and those patients who underwent one of the following procedures: (a) hemiarthroplasty or total hip replacement, (b) dynamic hip screw (DHS) fixation, and (c) cannulated screw fixation.

The exclusion criteria for case-control study included the following: patients scheduled for surgery but had conservative management instead, patients who died preoperatively, patients who underwent manipulation under anesthesia in the theatre, and those patients who were placed on palliative care instead of surgical intervention.

Cohort Study

The cohort component of the study focuses on patients who underwent hip fracture surgery between June 1, 2022, and June 1, 2023. We identified the patients who had perioperative hypotension and evaluated the outcomes over one year after surgery (from June 1, 2023, to June 1, 2024).

The inclusion criteria for the cohort study included the following: patients with perioperative hypotension who met the case-control inclusion criteria.

The exclusion criteria for the cohort study included the following: patients on end-of-life pathway, patients who did not mobilise on the first postoperative day due to inadequate pain control (for postoperative mobility outcome), patients who experienced immediate postoperative death (for 30-day morbidity outcome).

Declaration

We have used the abbreviation POH to only denote perioperative hypotension, and we declare that this does not include other similar terms like postoperative hypotension

Standard medical procedures in NOF fractures 

Patients with NOF fractures are usually operated on within 72 hours of admission. All the patients admitted to accidents and emergencies (A&E) with NOF fractures are transferred to the trauma ward. Most patients receive a fascia iliaca block in the A&E. All patients are discussed during the morning multidisciplinary team (MDT) meeting, which includes the trauma team, physiotherapists, and other relevant specialists. Following admission to the trauma ward, patients are monitored regularly using standard observations and the National Early Warning Score (NEWS) until they are deemed ready for surgery. Once anesthetic clearance is obtained, patients are transferred to the anesthetic room, where they receive spinal anesthesia or general anesthesia, depending on the clinical need. Blood pressure measurements are taken immediately preoperatively in both the ward and the anesthetic room. Intraoperative monitoring of blood pressure, including any episodes of hypotension, is conducted by the anesthesia team, with real-time data recorded in the hospital's electronic charting system. Postoperatively, patients are transferred to the recovery room, where they receive one-to-one nursing care for a minimum of four hours, with continuous monitoring to ensure stability. Subsequently, patients are returned to the trauma ward, where they are reviewed by senior house officers of the orthopedics department, to confirm clinical stability and ensure proper postoperative management.

POH definition

**Figure 1:**
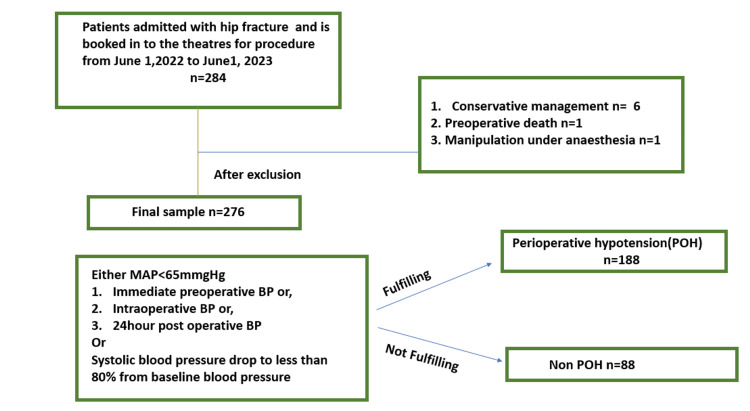
Perioperative hypotension study criteria POH: perioperative hypotension; MAP: mean arterial pressure; BP: blood pressure

POH is defined as a MAP below 65 mmHg at any point in the immediate preoperative, intraoperative, or postoperative period (24 hours after the surgery) or as a reduction in systolic blood pressure to less than 80% of baseline (Figure 1).

Risk factors and outcomes

The risk factors studied were grouped into three main categories: patient factors, anesthetic factors, and surgical factors. We studied the following risk factors: patient factors (age, gender, hypertension, diabetes mellitus, chronic kidney disease, heart disease (includes any history of ischemic heart disease, congestive heart disease, valvular heart disease, and hypertrophic obstructive cardiomyopathy), Charlson Comorbidity Index, preoperative hemoglobin (Hg), preinduction RBC values), anesthetic factors (ASA score, type of anesthesia), and surgical (perioperative) factors (type of operation, duration of surgery, postoperative Hg level, Hg drop (postoperative Hg-preoperative Hg), perioperative blood loss, and perioperative fluid).

Factors used to study perioperative blood loss- included documented blood loss in operation notes, preoperative and postoperative Hg, Hg drop (difference of immediate postoperative Hg and immediate preoperative Hg), and history of perioperative blood transfusion.

The outcomes studied included postoperative delirium, postoperative mobility, 30-day mortality, 365-day mortality, 30-day morbidity, and length of hospital stay.

Statistical analysis

Data was entered in MS Excel (Microsoft Corporation, Redmond, Washington, United States) and analysed using IBM SPSS Statistics for Windows, Version 25 (Released 2017; IBM Corp., Armonk, New York, United States). Categorical variables were expressed as frequency (percentage), and continuous variables were summarised using mean and standard deviation. Pearson's chi-squared test and Fisher’s exact test were used to evaluate the association of various categorical variables with the status of POH, and crude odds ratio (OR) with 95% confidence intervals were reported. Comparison of mean age across the perioperative hypotension status was done using independent t-test, while the Hg drop and ASA scores were compared using the Whitney U test. Relative risk (RR) based on POH for the development of various outcomes like delirium, mortality, morbidity, and length-of-stay postoperative mobilisation were calculated. Multivariate logistic regression analysis was employed to find the predictors for POH. For all these statistical interpretations, p-value < 0.05 was considered the threshold for statistical significance.

Ethical statement

According to the NHS Health Research Authority guidance, ethical approval was not required for this observational study. The project is registered as a quality improvement project (QIP) at Ashford and St Peter’s Hospitals NHS Trust. All information was collected following a formal request process.

## Results

Patient demographics and baseline characteristics

Out of 284 patients identified who were reported to have neck-of-femur (NOF) fractures during the study period, 276 were included in the study. The reasons for exclusion included the following: patients who underwent conservative management (n = 6), preoperative deaths (n = 1), and manipulation under anesthesia without surgery (n = 1). The incidence of POH is presented in Figure 1.

Risk factors

In the risk factor analysis, we studied patient factors, anesthetic factors, and surgical factors that can influence the expected outcomes of POH. Statistically significant risk factors found in the study were the history of hypertension (OR: 1.330) and chronic heart disease (OR: 2.768) (Table 1).

**Table 1:** Analysis of risk: patient and anesthetic factors POH: perioperative hypotension; OR: odd's ratio; CI: confidence interval; RBC: red blood cells;  ASA score: American Society of Anesthesiologists score *Statistically significant

Characteristics	Factors	Non-POH n (%)	POH n (%)	OR (95% CI)	p-value
Age group	≤80	31 (31.3%)	68 (68.7%)		0.894
	>80	57 (32.2%)	120 (67.8%)		
Gender	Male	27 (30.3%)	62 (69.7%)		0.783
	Female	61 (32.6%)	126 (67.4%)		
	Closed	6 (24%)	19 (76%)		
Hypertension	No	47 (39.2%)	73 (60.8%)	1.330(0.728-2.431)	0.027 ^* ^
	Yes	41 (26.3%)	115 (73.7%)		
Diabetes mellitus	No	77 (31.8%)	165 (68.2%)		1.00
	Yes	11 (32.4%)	23 (67.6%)		
Chronic kidney disease	No	77 (34.4%)	147 (65.6%)		0.071
	Yes	11 (21.2%)	41 (78.8%)		
Heart disease	No	76 (37.6%)	126 (62.4%)	2.768(1.273-6.071)	0.001 ^* ^
	Yes	12 (16.2%)	62 (83.8%)		
Charlson Comorbidity Index	≤5	67 (35.3%)	123 (64.7%)		0.094
	>5	21 (24.4%)	65 (75.6%)		
Pre-induction RBC (x10^12^/L)	≤4.13	38 (29.7%)	90 (70.3%)		0.518
	>4.13	50 (33.8%)	98 (66.2%)		
Preoperative haemoglobin (g/L)	<90	2 (28.6%)	5 (71.4%)		0.488
	90-120	19 (25.3%)	56 (74.7%)		
	121-150	64 (35%)	119 (65%)		
	>150	3 (27.3%)	8 (72.7%)		
ASA score	<3	24 (30.4%)	55 (69.6%)		0.777
	≥3	64 (32.5%)	133 (67.5%)		
Anaesthesia type	General	85 (31.8%)	182 (68.2%)		1.00
	Spinal	3 (33.3%)	6 (66.7%)		

Patients with hypertension (includes patients who have been treated or insufficiently treated) had a significantly higher incidence of POH (73.7%) compared to patients without hypertension (60.8%). The OR was 1.330 and is statistically significant, suggesting that hypertensive patients are 1.3 times more likely to develop POH (Table 1).

Patients with heart disease (ischemic heart disease, congestive heart disease, valvular heart disease, hypertrophic obstructive cardiomyopathy) were found to have a statistically significant higher incidence of POH in hip fracture surgeries. Patients with heart disease were found to have an OR of 2.76 with an incidence of POH at 83.8%, compared to 62.4% for those without heart disease (Table 1).

Other factors studied did not show a statistically significant association with a higher incidence of POH. Age > 80 years (67.8% vs 68.7%, p = 0.894), female vs male gender (67.4% vs 69.7%, p = 0.783), type of operation, closed vs open (76% vs 67.3%, p = 0.501), diabetes mellitus (67.6% Vs 68.2%, p = 1.00), type of anesthesia general vs spinal (68.2% vs 66.7%, p = 1.00); ASA score ≥ 3 ( 67.5 % vs 69.6%, p = 0.77), and pre-induction RBC > 4.13 (66.2% vs 70.3%, p = 0.518) (Table 1).

A higher Charlson Comorbidity Index, greater than 5, showed a higher incidence of POH (75.6%), compared to those with a score ≤ 5 (64.7%), but this association was statistically not significant (p = 0.094). Similarly, history of chronic kidney disease showed a significantly higher incidence of POH (78.8% vs 65.6%), although this was not statistically significant (p = 0.071) (Table 1).

Pre-induction hemoglobin (Hb) levels showed no significant association with incidence of POH. The incidence ratio did not suggest a relationship between high or low Hb levels. It was noted that the incidence of POH was higher when pre-induction Hb was 90-120 g/L (74.75) and that decreased incidence was found when Hb was below 90 g/L (71.4%) and above 150 g/L (72.7%). There was no statistical significance for these values, indicating that preoperative Hb levels were not strongly predictive of POH (Table 1).

Perioperative factors

We also studied the complications associated with this operation and the odds of the occurrence of POH with these factors (Table 2).

**Table 2:** Analysis of risk: perioperative factors POH: perioperative hypotension; OR: odd's ratio; CI: confidence interval *Statistically significant

Characteristics	Factors	Non-POH n (%)	POH n (%)	Crude OR (95% CI)	p-value
Operation type	Open	82 (32.7%)	169 (67.3%)		0.501
	Closed	6(24%)	19 (76%)		
Duration of surgery (hours)	<1 hour	26 (35.1%)	48 (64.9%)		0.770
	1-2 hours	58 (30.9%)	130 (69.1%)		
	>2 hours	4 (28.6%)	10 (71.4%)		
Postoperative hemoglobin (g/L)	<90	13 (31%)	29 (69%)		0.024^*^
	90-120	51 (27.7%)	133 (72.3%)		
	121-150	24 (48%)	26 (52%)	0.486 (0.206-1.145)	
Blood loss (mL)	≤200	45 (29.8%)	106 (70.2%)		0.275
	>200	37 (36.6%)	64 (63.4%)		
Blood transfusion perioperatively	No	80 (32.7%)	165 (67.3%)		0.542
	Yes	8 (25.8%)	23 (74.2%)		
Perioperative fluid (mL)	≤3000	59 (31.7%)	127 (68.3%)		1.000
	>3000	28 (31.8%)	60 (68.2%)		

Postoperative Hb levels showed a significant association with POH (p = 0.024, Fisher’s exact test) with limited strength of association. Patients with postoperative Hb levels above 120 g/L had the lowest incidence of POH (52.0%), while those with Hb below 90 g/L and those between 90 and 120 g/L had higher rates of hypotension, at 69.0% and 72.3%, respectively. The OR for hypotension in patients with postoperative Hb levels of 90-120 g/L was 1.169 (95% CI: 0.564-2.425), and for those with Hb levels above 120 g/L, the OR was 0.486 (95% CI: 0.206-1.145). These results suggest that higher postoperative Hb levels (particularly above 120 g/L) may be associated with a lower risk of POH and Hb above 120 g/L might be a protective factor for POH. We recognise the limitation of the total data and data distribution to further study this variable (Table 2).

When the duration of surgery was assessed, it showed a trend of increasing POH incidence with prolonged surgery. Surgery lasting less than one hour had a 64.9% incidence of POH, those with one to two hours had 69.1%, and those lasting more than two hours had the highest rate of 71.4%. Despite this trend, this was not statistically significant (p = 0.770) to be a predictor of POH (Table 2).

The comparison of Hb drop postoperatively (difference between preoperative and postoperative Hb) between the two groups revealed a statistically significant difference (Table 3). Patients who had POH had a mean drop of 20.98 g/L (SD: 13.570) with wide variability, compared to the patients without POH, who had a mean drop of 16.44 g/L (SD: 10.629). P-value was 0.004, and this factor was found to be significant.

**Table 3:** Analysis of risk: hemoglobin drop OR: odds ratio; CI: confidence interval ^*^Statistically significant

Factor	Perioperative hypotension	N	Mean (g/L)	Std. deviation	Mean rank	OR (95% CI)	p-value
Hemoglobin drop	No	88	16.44	10.629	118.50	1.042 (1.013-1.071)	0.004^*^
	Yes	188	20.98	13.570	147.86		

The complications such as blood loss, duration of surgery, perioperative blood transfusion, or perioperative intravenous (IV) fluid were found statistically negative. There was no significant association of giving perioperative fluid above 3000 mL IV (68.2% vs 68.3%, p = 1.00) with the outcome of POH. Blood loss and history of blood transfusion perioperatively were evaluated, and we found no strong association with POH. Patients with lower blood loss (≤200 mL) showed a higher incidence of POH (70.2% vs 63.4%, p = 0.275). Also patients who received blood transfusions perioperatively had a higher incidence of POH (74.2% vs 67.3%, p = 0.542) (Table 2).

We noted the discrepancy between the documented blood loss and the postoperative Hb levels. We concluded that this might be secondary to either the dilution of the postoperative sample due to collection methods or the inaccurate documentation of blood loss, as it is very subjective.

Outcomes

We studied immediate outcomes such as postoperative day of mobility, length of stay, postoperative delirium and long-term outcomes, 30-day morbidity, and 30-day or 365-day mortality (Table 4).

**Table 4:** Analysis of outcomes POH: perioperative hypotension; RR: relative risk; POD: postoperative day

Outcomes	Factors	Non-POH n (%)	POH n (%)	RR (95% CI)
Postoperative day mobilised	1^st^ day	34 (39.1%)	35 (19%)	Reference
	>1 day	53 (60.9%)	149 (81%)	1.329 (1.108-1.595)
Length of stay (POD)	<7 days	31 (35.2%)	32 (17.5%)	Reference
	≥7 days	57 (64.8%)	151 (82.5%)	1.273 (1.077-1.507)
Delirium	No	72 (81.8%)	118 (62.8%)	Reference
	Yes	16 (18.2%)	70 (37.2%)	2.037 (1.263-3.285)
30-day morbidity	No	81 (92%)	124 (66%)	Reference
	Yes	7 (8%)	64 (34%)	4.008 (1.944-8.262)
30-day mortality	No	81 (98.8%)	149 (92.5%)	Reference
	Yes	1 (1.2%)	12 (7.5%)	6.12 (0.81-46.19)
365-day mortality	No	81 (93.1%)	149 (84.7%)	Reference
	Yes	6 (6.9%)	27 (15.3%)	2.224 (0.954-5.186)

Postoperative patients are seen by physiotherapists and mobilised on day one if there is no complication or mobility restriction due to pain. In the cohort of 276 patients, POH was found to be associated with several clinically significant outcomes (Table 4).

After excluding five patients whose mobility was delayed due to pain, all other delay in mobility beyond postoperative day one were due to patients being symptomatically unwell. Among the POH group, 81% (n = 149/188) were not mobilised on day one, compared to only 60.9% (n = 53/88) in the non-POH group. This difference resulted in an RR of 1.32 and was statistically relevant (Table 4).

The length of stay was studied in terms of postoperative days, after excluding five patients who had prolonged stays for postoperative complications such as sepsis or due to immediate postoperative death. In the non-POH group, the mean length of stay was 7.37 days (SD: 3.25). The patients were studied as two groups: less than seven days of admission and more than seven days. POH was associated with prolonged hospital stay of more than seven days (RR: 1.27 (95% CI: 1.07-1.507)) (Table 4).

The incidence of delirium was notably higher among patients who experienced POH (37.2%), compared to the non-POH group (18.2%). The RR was 2.037, suggesting double the risk of having postoperative delirium for hypotensive patients (Table 4).

Mortality was analysed for two-time frames, 30 days and 365 days. Although both the mortality incidences were higher among the POH patients (30-day mortality: 7.5% vs 1.2%; 365-day mortality: 15.3% vs 6.9%), neither variable was statistically significant due to insufficient numbers.

**Figure 2:**
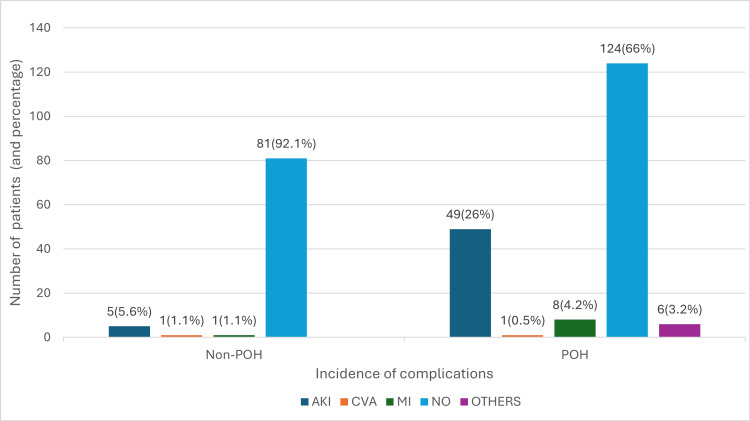
The 30-day comorbidities in the study population Non-POH: non-perioperative hypotensive group; POH: perioperative hypotensive group; AKI: acute kidney injury; MI: myocardial infarction; CVA:  cerebrovascular accident; Others: transient ischemic attack, pulmonary embolism, myocardial injury with acute kidney injury

POH was significantly associated with a 30-day morbidity with an RR of 4.008. The data showed an incidence of postoperative morbidity of acute kidney injury (AKI), myocardial infarction (MI), and cerebrovascular accident (CVA) of 34% among the hypotensive patients compared to 8% in the nonhypotensive group. The studied outcomes included AKI, MI, and CVA with the following distribution (Figure 2). The Others category (total n = 6) includes transient ischemic attack (TIA) (n = 1), postoperative pulmonary embolism (n = 4), and myocardial injury with AKI (n = 1) (Figure 2).

Intraoperative hypotension and duration

**Figure 3:**
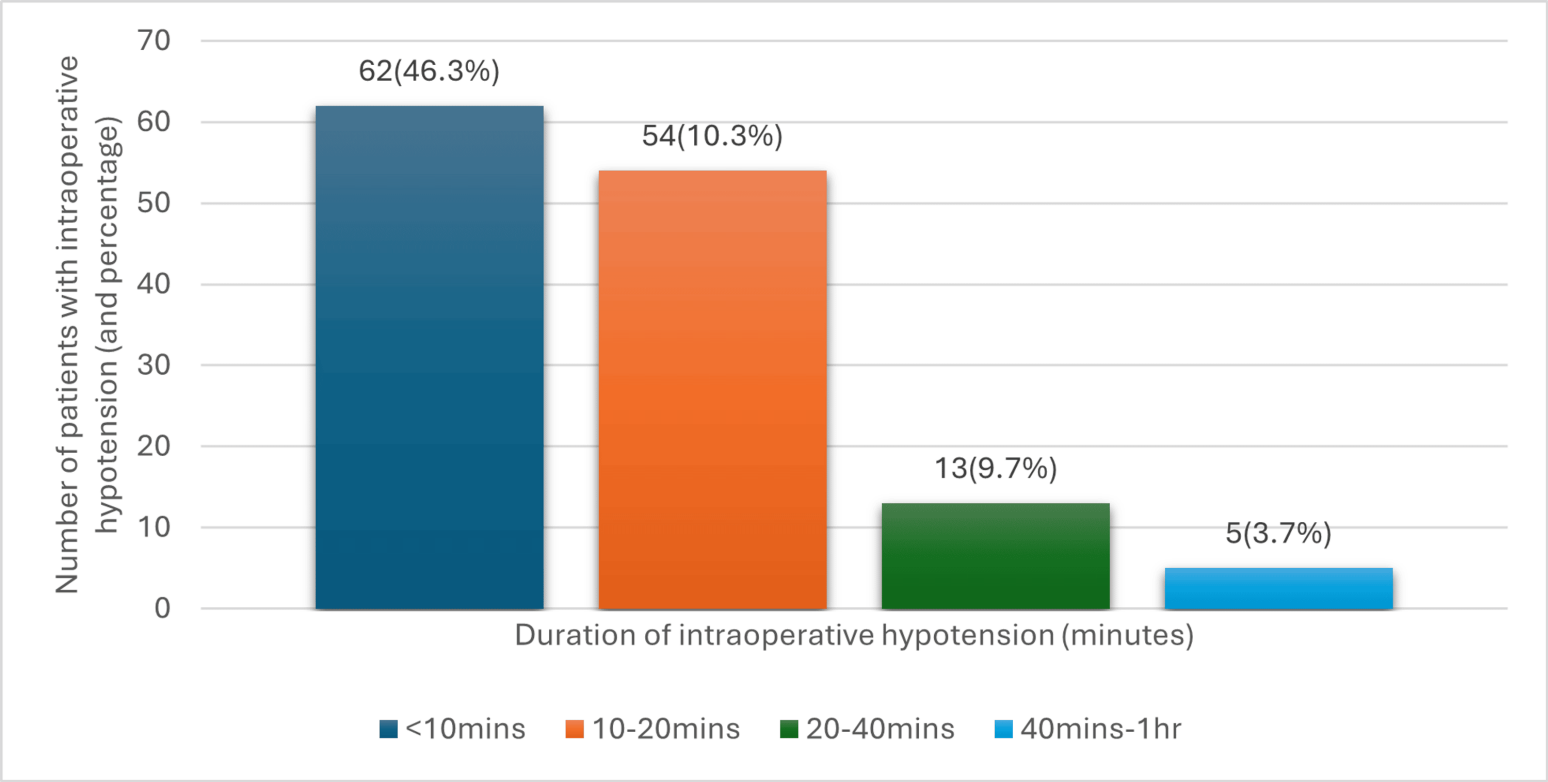
Duration of intraoperative hypotension in the study population mins: minutes; hr: hour Data of 134 patients who had intraoperative hypotension among 188 patients who had perioperative hypotension

Among 134 patients who had intraoperative hypotension, 46.3% had hypotension lasting under 10 minutes and 40.3% had hypotension lasting 10-20 minutes. A smaller proportion of patients (9.7%) had hypotension lasting 20 -40 minutes, and only 3.7% had hypotension lasting between 40 minutes and one hour (Figure 3).

**Figure 4:**
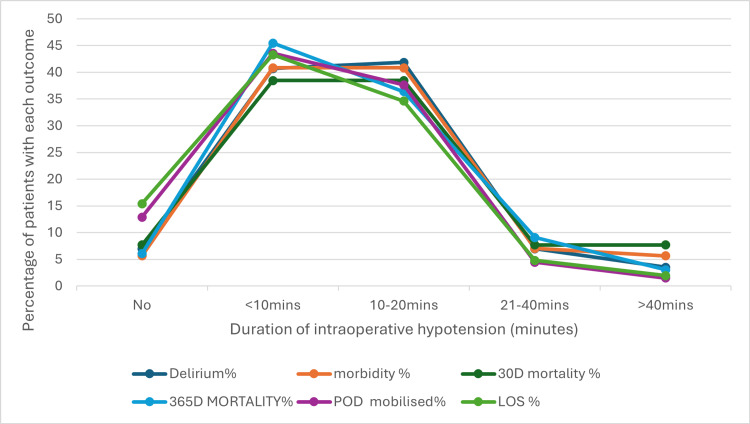
Relationship between duration of intraoperative hypotension and outcomes POD: postoperative day of mobilisation; LOS: length of stay The data shows the outcomes for 134 patients who had intraoperative hypotension, with the duration of their hypotensive episode

The study between the duration of intraoperative hypotension and postoperative outcomes revealed a complex, nonlinear association (Figure 4). The chart illustrates that the incidence of complications such as delirium, morbidity, and mortality peaks at 10-20 minutes of intraoperative hypotension, with all outcome percentages reaching their highest at this interval. Interestingly, as the duration extends beyond 20 minutes, the risk of adverse outcomes declines sharply, approaching levels seen in patients without hypotension. This bell-shaped curve indicates that the highest incidence of complication occurs during intermediate durations of hypotension, while both shorter and longer durations are associated with lower incidence. These findings underscore the importance of preventing hypotension, as its occurrence alone is a significant risk factor. While prolonged durations of postoperative hypotension do not show a consistent pattern of worsening outcomes, the study highlights that even short episodes of intraoperative hypotension pose substantial risks, emphasising vigilant management throughout the perioperative period (Figure 4).

Postoperative hypotension

**Figure 5:**
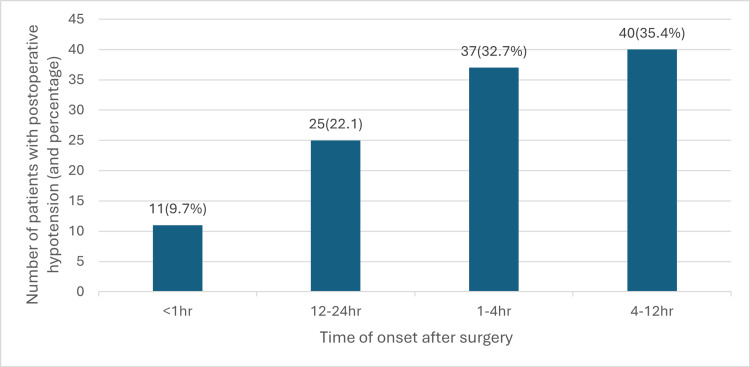
Relationship between the incidence of postoperative hypotension and the time of onset after the surgery hr: hours Data of 113 patients who experience postoperative hypotension, expressed in percentage against the time of onset since the surgery

The following tables provide an overview of postoperative hypotension (n = 113) with respect to the duration and timing from the start of surgery. The data showed that postoperative hypotension tends to occur within 1-12 hours after surgery. A combined total of 77/113 patients (68.14%) fall into this criteria. The likelihood of patients developing hypotension soon after surgery (less than an hour) is under 10% (Figure 5).

**Figure 6:**
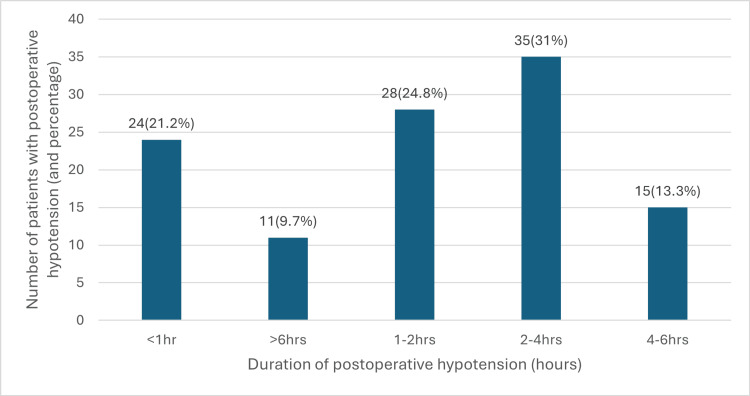
Duration of hypotension in patients who experienced postoperative hypotension hrs: hours Data of duration of hypotension in patients who had postoperative hypotension (n = 113) expressed as percentage

The graph plotted for patients who had postoperative hypotension and the duration of the episode showed that out of the 113 patients who had postoperative hypotension, only about 46% (n = 52) were managed within two hours of onset of hypotension. The 10% of patients had persistent postoperative hypotension lasting more than six hours, most occurring overnight. A separate study is required to investigate the factors related to delayed management or persistent hypotension in surgical wards (Figure 6).

**Figure 7:**
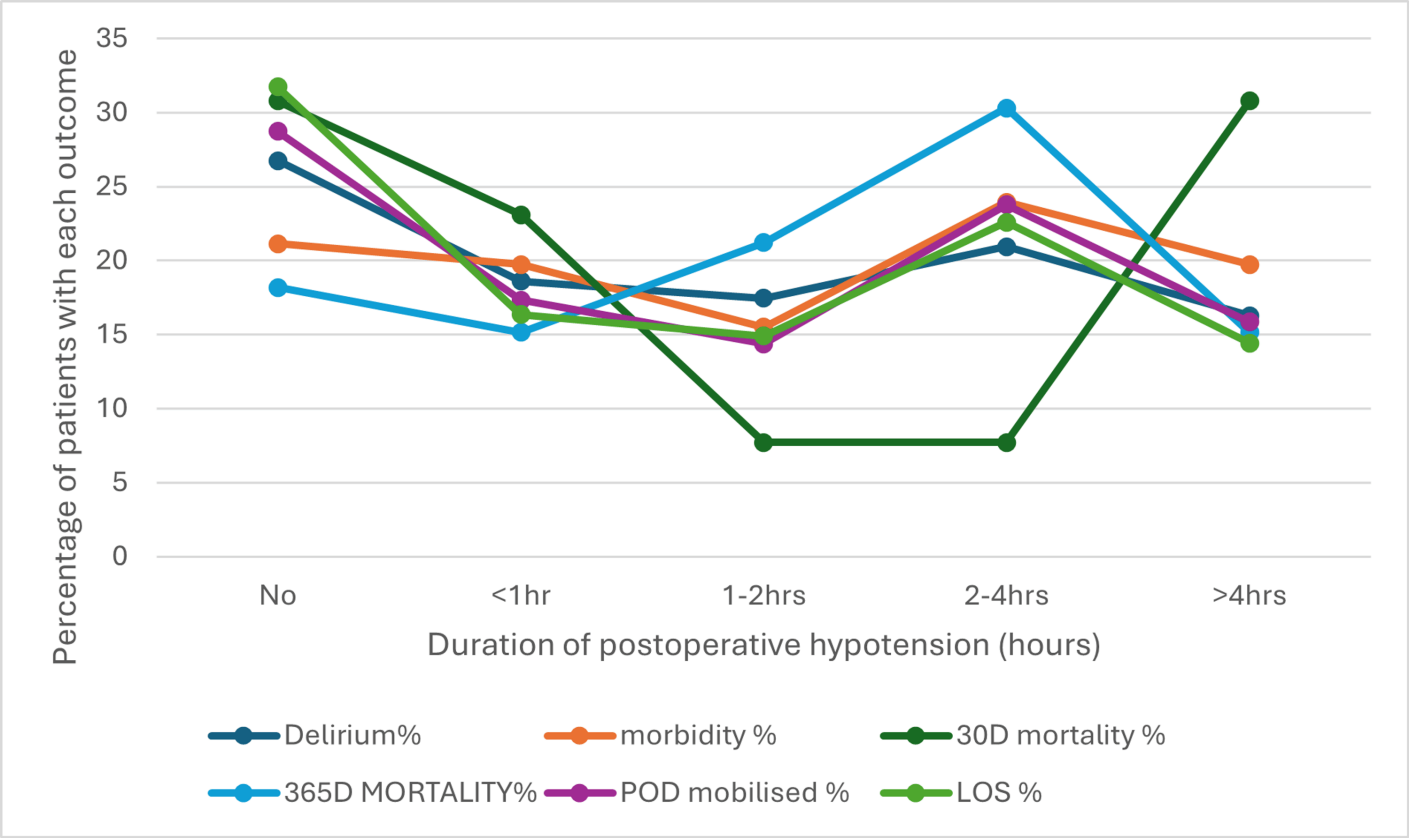
Relationship between the duration of postoperative hypotension and outcomes POD: postoperative day of mobilisation; LOS: length of stay; hr(s): hour(s) The data shows the outcomes for 113 patients who had postoperative hypotension, with the duration of their hypotensive episode

The relationship between postoperative hypotension and various outcomes revealed a complex and unpredictable pattern (Figure 7). For hypotension lasting less than one hour, the incidence of complications remains low. As duration of hypotension tends to rise beyond one to two hours, a notable spike in the 30-day mortality and a general increase in the 365-day mortality were observed. However, the incidence of the 365-day mortality decreases beyond four hours, which is not clinically explainable. Other complications such as delirium, morbidity, postoperative mobilisation, and length of stay rise moderately with duration but follow no predictable pattern. While there is a general tendency for complications to increase with longer durations of hypotension, the absence of a clear pattern highlights the unpredictable nature of these risks.

## Discussion

In this study, we identified key risk factors for POH among elderly patients undergoing hip fracture surgery, including a history of hypertension, chronic heart disease, and perioperative blood loss. These factors increase the susceptibility of this population to hemodynamic instability, which is associated with multiple adverse outcomes, as demonstrated in our findings. These outcomes include an increased risk of delirium, prolonged hospital stays, delayed postoperative mobility, and higher 30-day and 365-day mortality, as well as increased 30-day morbidity.

Hip fracture is a critical injury and a major cause of admission in trauma wards within the elderly population. As per the NHFD data from 2023, a total number of 69,375 cases of hip fractures were reported in UK hospitals. The injury usually stems from multiple factors. Given that hip fractures mostly affect the elderly population, there is an associated increased risk of mortality and morbidity linked to them [6].

The lack of a well-defined explanation of the criteria for diagnosing POH needs to be addressed before delving further into the study. A precise, evidence-based definition was necessary, as there is no universally defined target range for intraoperative blood pressure [2].

The criteria used in previous studies were reviewed, and the most commonly adopted definitions include a decrease in MAP below 65 mmHg and a decrease in systolic blood pressure to less than 80% of the baseline [2-5,7]. MAP is a particularly reliable indicator of blood pressure as it reflects cardiac output and downstream arterial resistance, making it a good marker for organ perfusion [9]. Saugel and Sessler emphasize that while classifying patients based on hypotensive criteria, the duration and severity of hypotension must also be considered essential factors [4].

Our study showed a clear association between POH and increased postoperative complications, though the relationship was complex and nonlinear across both intraoperative and postoperative periods. These results highlight the need to develop preventative strategies for POH and the imperative of promptly addressing even short episodes of hypotension, as such episodes are potential risk factors for adverse outcomes. As Ince et al. describe, even brief exposure to hypotension can disrupt microcirculation leading to tissue damage [10]. Once systemic blood pressure is restored, hemodynamic incoherence can occur and result in persistent tissue hypoxia due to deficient microcirculation [10]. This aligns with our study findings that short duration of hypotension was linked to complications, underscoring the importance of immediate management.

Given these insights, it is essential to discuss the multifactorial causes of intraoperative hypotension. Patient-related factors, such as advanced age, are themselves risk factors due to a decrease in physiological reserve [11,12]. Age-related pharmacokinetic and pharmacodynamic differences in elderly increase their susceptibility to the possible complications from anesthetic agents, one of which can be hypotension [13]. Additionally prolonged surgeries can lead to vasodilation, which in turn can cause hypotension [14].

Our case-control study identified hypertension, history of heart disease, and postoperative blood loss as significant risk factors for developing POH. Previous literature has shown that before the advent of blood pressure treatments, a blood pressure above 200/110 mmHg was linked to cardiovascular complications during anesthesia and surgery [15]. Additionally, the impact of antihypertensive therapy may also predispose patients to intraoperative hypotension, as evidenced by the association between renin-angiotensin system (RAS) inhibitors and hypotension [11]. However, the 2024 SPACE trial advises against stopping RAS inhibitors, as evidence does not support a reduced incidence of myocardial injury with their discontinuation. In our study, only four patients met the criteria for severe hypertension preoperatively (blood pressure above 180/120mmHg as defined by the National Institute of Health and Care Excellence, Clinical Knowledge Summaries (NICE CKS)) [16], and interestingly, all four experienced intraoperative hypotension, reflecting findings in prior literature. Furthermore chronic hypertensive patients experience a right-sided shift in autoregulatory curves, which makes them susceptible to hypotensive complications at a lower threshold than normotensive patients [4].

A history of heart disease was also identified as a significant risk factor, with an odd’s ratio of 1.273, showing an 83.8% incidence of POH in affected patients. A previous study has corroborated that any history of ischemic heart disease or congestive heart disease limits cardiac reserve, which predisposes patients to have POH [12]. Yang et al. demonstrated that left ventricular dysfunction in patients undergoing hip fracture surgery can result in pulmonary congestion and acute heart failure after fluid resuscitation for managing hypotension [7]. The Revised Cardiac Risk Index score (RCRI), a predictor of perioperative risk in patients with cardiac disease history, undergoing noncardiac surgery has been identified as a risk factor for postoperative cardiac and renal complications [12]. The Canadian Cardiovascular Society has recommended using the RCRI score to assess cardiovascular risk in patients aged ≥45 years or others with significant cardiovascular disease who undergo elective noncardiac surgery; patients in the risk category should be frequently monitored for outcomes [17]. Further research is necessary to explore the potential of this score in targeted monitoring.

Our findings also support perioperative blood loss as a risk factor for hypotension. We assessed quantitative blood loss (as documented in the operation notes), history of perioperative blood transfusion, postoperative Hg, and Hg drop (difference between pre- and postoperative values). Despite potential discrepancies due to subjective documentation, our study showed a significant positive association between Hg drop and POH. While we acknowledge the potential for errors in documenting blood loss, we ruled out the possibility of sample dilution in post-Hg measurements by comparing the results with those from subsequent days. Our study found a mean Hg drop of 20.98 g/L in the POH group compared to 16.44 g/L in the non-POH group. This corresponds to the findings of Kumar et al. in hip fractures where the average drop in Hg was shown to be 2.23 g/dl to 0.7 g/dL (22.3g/L to 7g/L) [18]. Our results for postoperative Hg were statistically significant, with a maximum incidence of POH occurring in the postoperative Hg range of 90-120g/L. However, it showed a protective effect with a crude odd’s ratio of 0.49, with Hg above 120g/L. It should be noted that preinduction Hg range showed that maximum incidence of POH is in the range of 121-150g/L. This echoes the results from the above study that preoperative Hg is misleading and can lead to very low postoperative Hg levels [18]. Regular perioperative Hg monitoring is recommended in hip fracture surgeries to monitor the perioperative hidden blood loss [19]. Perioperative transfusion in patients has not shown significance in the current study of incidence of POH, and Carson et al. reported insufficient evidence to prove that transfusion improved complications in hip fracture surgery [20]. The surgical APGAR score, which only uses intraoperative parameters such as intraoperative blood loss, lowest heart rate, and lowest MAP showed moderate discriminative ability to predict postoperative morbidity [2,21].

The results of the cohort study on the outcomes of POH were noteworthy. Patients with POH experienced higher rates of delirium, 30-day morbidity, prolonged length of stay, and delayed mobilisation. Hemodynamic instability likely contributes to these complications and can be explained in light of the autoregulation of vital organs. The autoregulatory range for cerebral perfusion ranges from 60 to 160 mmHg, with higher thresholds in hypertensive patients [2]. In the study done by Joshi et al., the lower limit of autoregulation (LLA) was around 66 mmHg of MAP (95% prediction interval: 43-90 mmHg), and this threshold varies among individuals [22].

MAP is the principal inflow pressure for most organ systems and is significant in cerebral perfusion and renal perfusion [4]; however, cardiac perfusion depends more on diastolic blood pressure [23]. Anesthetic drugs can further impair these functions, risking end-organ damage.

Among the 30-day morbidity, AKI was the most frequent, occurring in 26% of POH patients, which aligns with findings by Lankadeva et al. in their review [24]. Cardiac complications including myocardial injury are seen in POH, particularly in patients with high pulse pressure prior to anesthesia [25]. This poses an immediate risk factor for postoperative mortality [4]. It may be concluded that the organs which are highly autoregulated are more vulnerable to injury secondary to in the blood pressure fluctuations [9].

Postoperative delirium occurs frequently in association with POH in the elderly population, but it is often missed. The cognitive function of the brain diminishes with age, and it adds to the inherent risks of hypotension, which are further influence by fluctuations in the blood pressure [26]. Our study showed significant incidence of delirium in hypotensive patients after surgery, and there was a significant risk factor of 2.037 (1.263-3.285).

POH has been regarded as a risk factor for mortality in different studies previously [7,27]. Our study showed a 30-day mortality incidence of 7.5% in patients with POH, which corresponds to the 9.6% reported in the literature [28]. This is consistent with the NHFD of our hospital, where crude mortality was at par or below the national average during the study period (June 2022-June 2023) (https://www.nhfd.co.uk/20/nhfdcharts.nsf/vwcharts/Mortality?open&org=SPH). The 365-day mortality in our study was 15%, which again was lower than that described by Roche et al. [28].

Said et al. described that day one mobilisation is often delayed due to hypotension [29], and accordingly, we have also found that mobility was delayed for 81% of the patients with hypotension, likely due to symptomatic postural blood pressure drop during mobilisation or patients feeling too unwell to move. Delays in postoperative mobilisation eventually lead to longer hospital stays and delayed rehabilitation. In the review by Wesselink et al., it was reported that three studies identified prolonged hospital stays, while two other studies reported median number of days of hospital stay between four and seven days. In our study, the mean length of stay in the nonhypotensive group was less than seven days. Moreover, 82.5% of patients in POH group remained hospitalised for more than seven days compared to 64.8% in the nonhypotensive group [30]. This warrants a prompt management of the POH.

In acknowledging the limitations of this study, we realize that lack of a clear, universally accepted definition of POH presents a challenge. We have reviewed the existing literature and adopted the most commonly used definition. Additionally, we understand that interpreting hypotensive readings alongside vasopressor use could have provided more insight into the incidence of clinically relevant hypotension episodes. Further research is required to explore this aspect. In our study, we recognize that insufficient mortality data limits strong statistical conclusions. Increasing the sample size is recommended to strengthen the data for future studies. Although postoperative Hg levels were statistically significant without notable clinical implications, increased Hg levels appear protective. The management of POH is crucial; there is also a need for continued research and strategies to address the current issues.

## Conclusions

This study demonstrates that POH is strongly associated with adverse outcomes, including increased risk of delirium, prolonged hospital stays, delayed mobility, and elevated 30-day morbidity and mortality in elderly hip fracture patients. Key risk factors for POH include a history of hypertension, chronic heart disease, and perioperative blood loss. Targeted assessment and intensive monitoring of high-risk patients, along with proactive management of hypotensive episodes, are essential to mitigate these risks and improve surgical outcomes. Early intervention to address hypotension is critical in preventing long-term complications and optimising recovery in this vulnerable population.
